# Modeling the head of PRIMUS linear accelerator for electron‐mode at 10 MeV for different applicators

**DOI:** 10.1002/acm2.12836

**Published:** 2020-02-18

**Authors:** Hani Negm, Moamen M. O. M. Aly, Walaa M. Fathy

**Affiliations:** ^1^ Physics Department College of Science Jouf University Sakaka Saudi Arabia; ^2^ Physics Department Faculty of Science Assiut University Assiut Egypt; ^3^ Radiotherapy and Nuclear Medicine Department South Egypt Cancer Institute Assiut University Assiut Egypt

**Keywords:** applicator, BEAM_NRC_, EGS_NRC_, electron beam, percentage depth dose, primus linear accelerator

## Abstract

**Objective:**

This study is to validate the utilization of Monte Carlo (MC) simulation to model the head of Primus linear accelerator, thereafter, using it to estimate the energy fluence distribution (EFD), the percentage depth dose (PDD), and beam profiles.

**Materials and Methods:**

The BEAM_NRC_ code that is based on the EGS_NRC_ code has been used for modeling the linear accelerator head for 10 MeV electron beam with different applicator sizes (10 × 10, 15 × 15, and 20 × 20 cm^2^). The phase space was acquired from BEAM_NRC_ at the end of each applicator and then used as an input file to DOSXYZ_NRC_ and BEAMDP to calculate the EFD, PDD, and beam profiles.

**Results:**

There were a good consistency between the outcomes of the MC simulation and measured PDD and off‐axis dose profiles that performed in a water phantom for all applicators. The PDD for the applicators proved to be favorable as a direct comparison of R_100_, R_90_, R_80_, and R_50_ yielded results of < 2 mm, while it was 6 mm in R_100_ for the applicator 15 × 15 cm^2^. The discrepancies in the surface doses (<3%) showed a quick decline in the build‐up region and differences reached 0% within the first 2.4 mm. For the beam profiles comparison, the differences ranged from 2% (2 mm) to 3% (6 mm) for all applicators.

**Conclusion:**

Our examination demonstrated that the MC simulation by BEAM_NRC_ code was accurate in modeling the Primus linear accelerator head.

## 1 INTRODUCTION

The fundamental modalities of malignancy treatment are radiotherapy, chemotherapy, and surgery.^1^ The treatment modality is usually chosen based on the stage and type of disease. Over 40% of all cancer sufferers are treated with radiation treatment whereby a therapeutic dose of ionizing radiation is conveyed to a malignancy site in the expectation of killing tumor cells. The objective of radiation treatment is to kill tumor cells by causing irreparable damage to their DNA while sparing normal cells as meager harm as possible.^2^ There are several machines in use for radiotherapy cancer treatment, yet linear accelerator (LINAC) based radiotherapy is the most common used machine worldwide. Deep‐seated tumors are usually treated by x‐rays produced by bremsstrahlung interaction of electron beam with a target. However, superficial tumors are usually treated by electron mode of a LINAC.^3^

While the limited scope of electrons in tissue has restorative advantages in radiation treatment including electron beams, prediction of dose for electron beams incident on heterogeneous tissue can be challenging in radiation treatment plannin.^4^ A uniform ‘plateau’ of dose could be delivered by a single electron beam, ranging from 90% to 100% of maximum central axis dose, in which the dose suddenly falling off both laterally and distally. This has allowed superficial cancers and disease within 6 cm of the patient's surface to be irradiated with low dose to underlying normal tissues and structures, something usually not possible with x‐ray therapy.^5^ Electron beams have been successfully used in numerous sites such as head and neck to avoid irradiation for spinal cord. It is also used for chest wall radiotherapy to avoid excessive irradiation of lung.^6^ The complex nature of electron tissue interactions means that electron beams are generally difficult to model. In electron beam therapy, calculation of collimator scatters and leakage, prediction of dose in small fields, situations involving sudden changes in surface contours, small inhomogeneities, and oblique beam incidences are particularly challenging.^7^ Monte Carlo (MC) simulation is a precise and specified method of modeling the complex electron source configurations and geometries used in radiation therapy. It is known to be very accurate when used properly for patient‐specific dose calculations.^8^, ^9^ Monte Carlo simulation can give an extensive variety of accurate data, including data which is difficult or impossible to quantify.^10^ A portion of the early employment of the MC method included estimations of mass stopping power ratios and the relationship between mean energy at the phantom surface and the practical range of the electron beam as recommended for electron beam dosimetry by ICRU Report 35.^11^, ^12^ Monte Carlo can possibly unravel a significant number of electron transport problems, especially in‐patient heterogeneities, encountered with conventional treatment planning algorithms.^13^ The principal disadvantage of MC simulation as applied to radiation transport has been the long computation time. The development of faster MC codes and enhancements in computers processor speeds have significantly reduced computation time. Today all codes of practice for absolute dose calibration use MC derived water to air stopping power ratios, S_water,air_ 14, 15 and additionally, several commercial vendors have started to receive MC algorithms for electron treatment planning.^16^, ^17^

Simulation of the treatment head of linear accelerators utilizing a detailed description of the head geometry and components has become an important aspect of dose computation in radiation therapy. The accuracy of the model is usually assessed by comparison of measured (in water phantom) and MC calculated beam parameters such as output factors and dose distributions. MC treatment head simulation has few control parameters, making calculations highly sensitive to errors in the beam characterization. For this reason, it is important to be aware of the sensitivity of MC simulation results to details of the initial electron beam (source), geometry of the treatment head and the necessity for accurate measured data. For example, the electron beam range (R_50_) in water is highly sensitive to the initial electron energy (0.1 cm change per 0.2 MeV) and the source energy is, therefore, the primary tuning parameter in electron beam simulations. However, electron beams are also very sensitive to all components in the beam path and therefore accurate geometric descriptions of all treatment head components is required.^18^

Monte Carlo simulations of radiation treatment machine heads provide practical means for obtaining energy spectra and angular distributions of photons and electrons. So far, most of the work published in the literature has been limited to photons and the contaminant electrons knocked out by photons.^19^ The dimensions and materials used in various components in the machine head (e.g., primary collimator, flattening filter, etc.) are specified as input to the code. Therefore, a different accelerator can easily be described by modifying these inputs.^20^ To confirm the validity of the energy spectra and angular distributions generated by the MC programs, one may calculate dose distributions using these data, and compare the results of calculations with measured depth dose data.^21^

We aimed in this study to simulate the electron mode of Siemens Primus linear accelerator at energy 10 MeV for different applicator sizes 10 × 10, 15 × 15, and 20 × 20 cm^2^. Then compare the simulation calculation of the percentage depth dose and dose profiles with the corresponding data acquired by measurements.

## 2 MATERIALS AND METHODS

### 2.1 Medical linear accelerator

**Figure 1:**
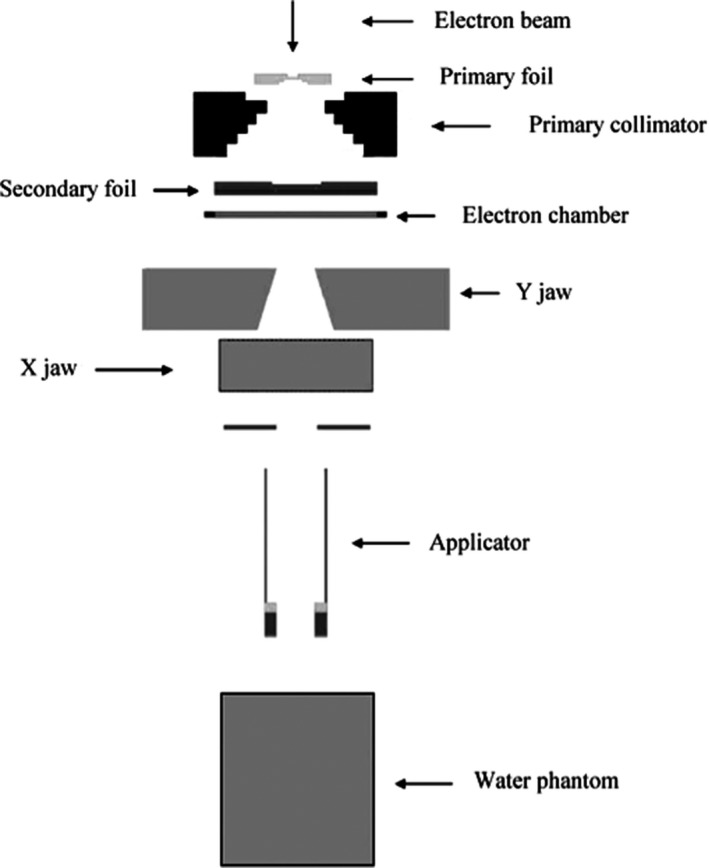
Geometry of head of Siemens Primus LINAC in electron mode as well as the water phantom 22

All experimental measurements and MC simulations were performed on the medical Siemens Primus linear accelerator (LINAC) which installed in the South Egypt Cancer Institute Center (SECI). This LINAC provides two nominal photon energies: 6 and 15 MV as well as six nominal electron energies: 5, 7, 8, 10, 12, and 14 MeV. The electron beam energy with 10 MeV has been studied to validate the BEAM_NRC_ for the dose calculations. MC simulation was based on the geometry of the head components of the LINAC in electron mode which consists of primary scattering foil, primary collimator, secondary scattering foil, dose monitor chamber, X and Y jaws, and applicator. These foils are made from different materials with different thicknesses. The primary collimator made of stainless steel, the secondary scattering foil made of aluminum, the ion chamber made of Kapton, and the applicators made of aluminum. The geometry of the LINAC head is illustrated in Fig. 1.

### 2.2 Experimental measurements

For relative experimental data such as PDD and off‐axis profiles, two calibrated simple ionization chambers (Semiflex 31010, PTW‐Freiburg, Germany) which has an inner volume of 0.125 cm^3^, one used as a sample detector and the other used as a reference detector, were used. The sample detector was accurately aligned to the beam central axis and the chamber's effective point of measurement was set at the water surface. A 3D water tank dosimetry system (DynaScan, CMS Associates int., USA) was used to control the chamber positioning and collect the measured ionization. The collected ionization signals are passed to a preamplifier before reaching the operator computer which reads and draws the ratio between these two signals. The collected percentage depth ionization curves were then converted to the corresponding PDD curves according to TG‐51 and the latter were used in the comparisons. PDD and dose profile were measured for 10 × 10 cm^2^, 15 × 15 cm^2^, and 20 × 20 cm^2^ applicator sizes.

### 2.3 Monte Carlo simulation

The MC simulation was performed by using BEAM_NRC_ code which based on EGS_NRC_ simulation package. It is a MC code that utilizes EGS_NRC_ and can be utilized to simulate the transport of radiation through a virtual linear accelerator model. The EGS (Electron Gamma Shower) arrangement of PC codes is a universally useful bundle for the MC simulation of the coupled transport of electrons and photons in an arbitrary geometry for particles with energies above a few keV up to several hundreds of GeV.^23^ BEAM_NRC_ is a MC simulation code for modeling radiotherapy sources.^24^ It accompanies a library of so‐called component modules. These are the basic parts used to build the accelerator. Using BEAM_NRC_ to model any linear accelerator head starts by defining the component modules and their functions. Then, define their dimensions, materials, and correct positions to make the model as accurate as possible. The component modules which were utilized in BEAM/EGS_NRC_ were as the follow: FLATFILT for the primary scattering foil, primary collimator, secondary scattering foil, CHAMBER for the monitor chamber, JAWS for the secondary collimator and APPLICAT for the applicator (Fig. 1). PRESTA (Parameter Reduced Electron Step Transport Algorithm) is introduced into the EGS code system to improve the accuracy of modeling of electron transport.^25^ We picked EXACT boundary crossing algorithm (BCA) with the goal that electrons are transported in single elastic scattering mode as soon as they reach a distance from the boundary defined by the skin depth for BCA. The default value of three mean free paths is recommended to give peak efficiency. Table 1 presents the main parameters of the EGS_NRC_ simulation that were used in our calculations.

**Table 1:** EGS_NRC_ main parameters that are implemented in the study

Maximum step‐size (S_max_)	1 × 10^10^
Maximum fractional energy loss/step (ESTEPE)	0.25 cm
XImax	0.50
Boundary Crossing Algorithm (BCA)	Exact
Skin depth for BCA	3 MFP
Electron Step algorithm	PREATA II
Spin effects	Off
Electron impact ionization	Off
Bremsstrahlung angular sampling (IBRDST)	Simple
Bremsstrahlung cross‐section	Bethe Heitler (BH)
Bound Compton scattering	Off
Compton cross‐section	Off
Pair angular sampling	Simple
Pair cross‐section	Bethe Heitler (BH)
Photoelectron angular sampling	Off
Rayleigh scattering	Off
Atomic relaxations	Off
Photon cross‐section	Si
Photon cross‐section output	Off

An electron source with a diameter of 1 mm was chosen with the direction downward toward the phantom surface. Source‐surface distance (SSD) was set as 100 cm. The electron beam source was demonstrated by ISOURC = 0 module which was a parallel beam of the front. We utilized transport parameters, for example, E‐CUT, P‐CUT which are utilized to characterize the global electron and photon cut‐off energies, were set to 0.521 and 0.01 MeV respectively. No electron range rejection techniques were used. For more precision, the number of histories was selected as 1 × 10^7^ for each simulation. Default values were utilized for the parameters reduced electron step transport algorithm (PRESTA‐II) in all simulations. Table 2 presents the main parameters of the BEAM_NRC_ simulation that were used in our calculations.

**Table 2:** BEAM_NRC_ main input parameters that are implemented in the study

Number of histories	1 × 10^7^
Random number seed 1	33
Random number seed 2	97
Bremsstrahlung splitting	None
Bremsstrahlung cross section enhancement	Off
Global electron cut‐off energy (ECUT)	0.521 MeV
Global photon cut‐off energy (ECUT)	0.01 MeV
Electron range rejection	Off
Photon forcing	Off

At the bottom of the applicator, the phase‐space file was created. This phase‐space contains information about position, direction, energy, and charge for each particle passing this level. At that point, this phase‐space utilized as input file in DOSXYZ_NRC_ user code which is a general purpose MC EGS_NRC_ user code for three‐dimensional absorbed dose calculations and simulates the transport of photons and electrons in a Cartesian volume and scores the energy deposition in the designated voxels.^26^ The phase‐space at SSD = 95 cm was used to obtain off‐axis dose profiles at R_100_ with source surface distance (SSD) of 100 cm in the water phantom of 25.6 × 25.6 × 25.6 cm^3^ with voxel size 0.2 × 0.2 × 0.2 cm^3^. The size of the water phantom was chosen as the default maximum number of voxels in the DOSXYZ_NRC_ is 128 voxels. Finally, the simulated PDD and dose profiles were compared with the corresponding measured data with normalization to the depth of maximum dose in order to validate our MC simulation. Table 3 presents the main parameters of the DOSXYZ_NRC_ simulation that were used in our calculations. The BEAMDP user code was used in analyzing the electron beam data obtained by the MC simulation of the coupled transport of photons and electrons such as derive energy fluence distribution^27^ for both photons and electrons components of the beam.

**Table 3:** DOSXYZ_NRC_ input parameters that are implemented in our work

Incident particle	All
Number of histories	1.2 × 10^8^
Random number seed 1	33
Random number seed 2	97
ECUT	0.521 MeV
PCUT	0.01 MeV
Range rejection	Off
Medium surrounding phantom	Air
Incident beam size	25
NRCYCL	0
HOWFARLESS	On

## 3 RESULTS

**Figure 2:**
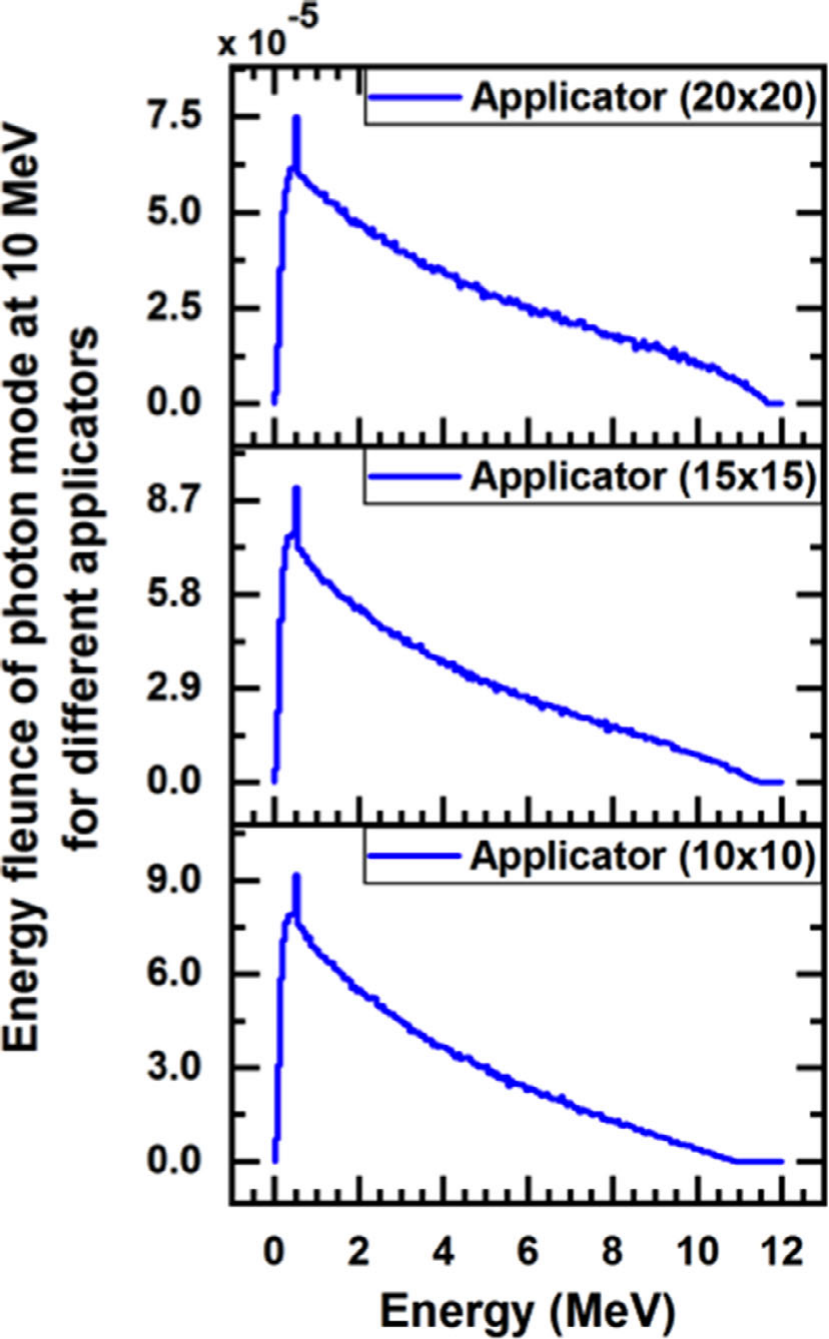
Calculated Monte Carlo energy‐fluence of the photon for different applicators with 10 MeV nominal‐energy

**Figure 3:**
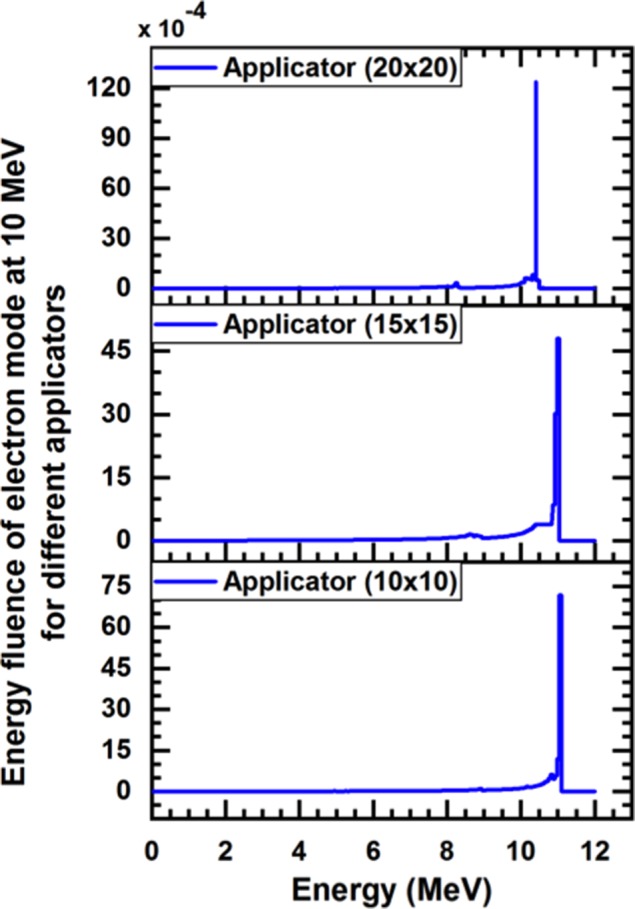
Calculated Monte Carlo energy‐fluence of the electron for different applicators with 10 MeV nominal‐energy

**Figure 4:**
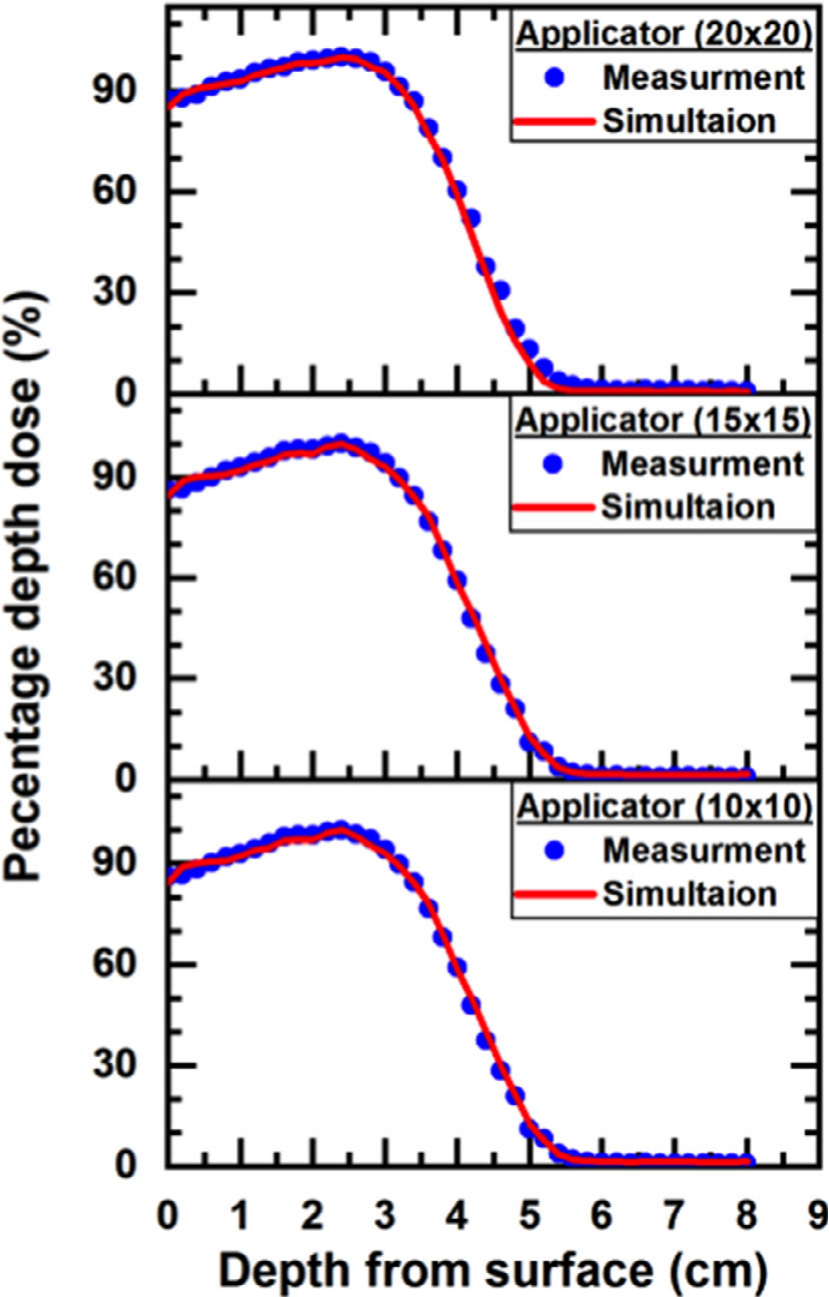
The Measured and the Monte Carlo‐calculated percentage depth dose for different applicators with 10 MeV nominal energy

Figures 2 and 3 presented the energy spectra of photons and electrons for 10 MeV of the incident particle for different applicators sizes (10 × 10, 15 × 15, and 20 × 20 cm^2^) at the phantom surface (SSD = 100 cm). The phase‐space that acquired by BEAM_NRC_ was used as an information document to BEAMDP to derive energy‐fluence distribution. Table 4 presents the total number of the particles which exit from the end of each applicator, number of photons, number of electron (positron), and the maximum kinetic energy for the nominal energy 10 MeV. The variation of PDD curves of the various electron beams for different applicator sizes (10 × 10, 15 × 15, and 20 × 20) cm^2^ were shown in Fig. 4.

**Table 4:** The total number of the particles that exit from the end of each applicator; summary of particles number in the phase‐space file

Applicator (cm^2^)	Total number of particles	Total number of photons	Photons (%)	Electrons (Positrons) (%)	Max kinetic energy (MeV)
10 × 10	2 289 746	1 562 182	68.0	32.0	11.438
15 × 15	3 355 066	2 092 171	62.4	37.6	11.454
20 × 20	4 575 656	2 368 872	51.8	48.2	11.463

**Figure 5:**
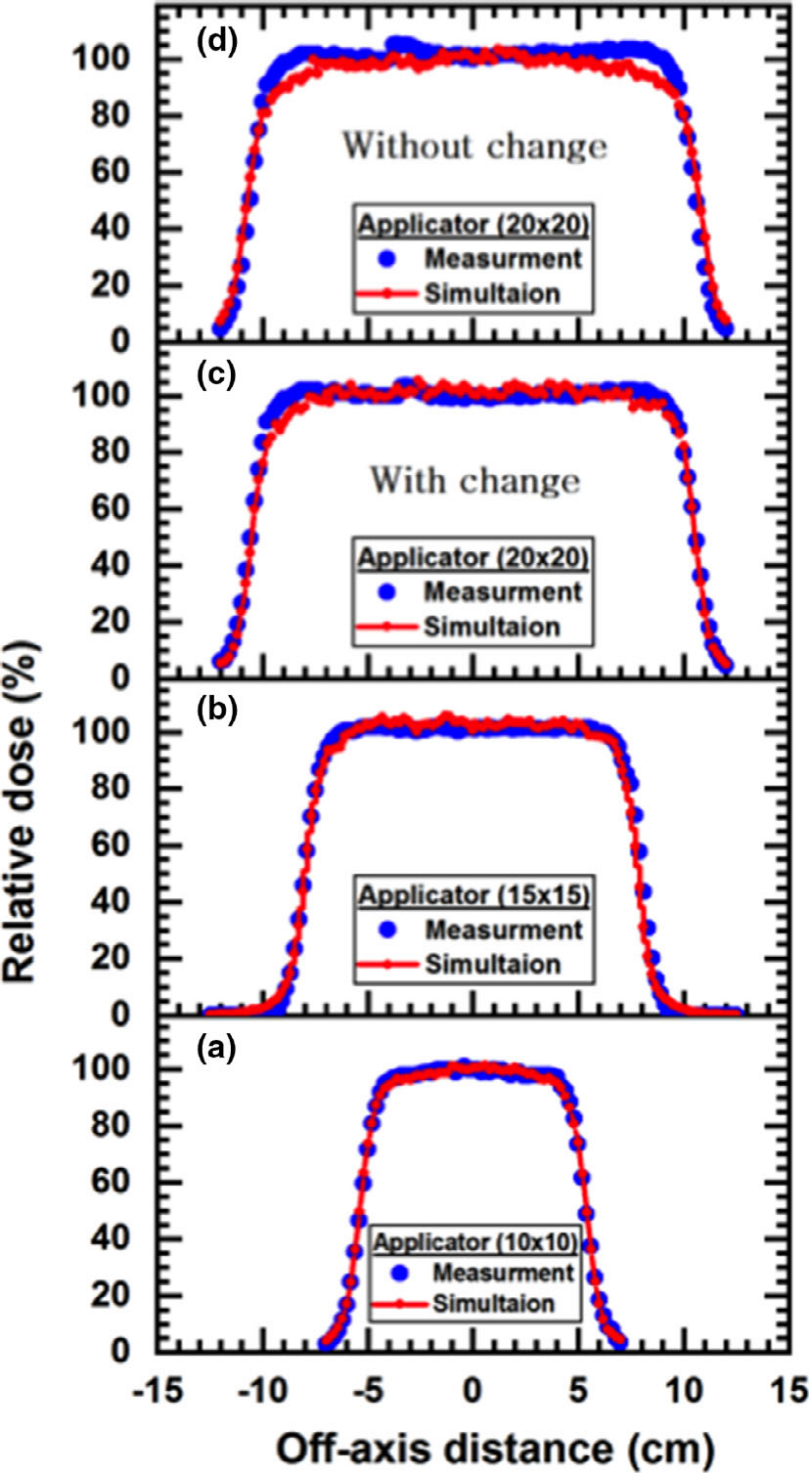
The Measured and the Monte Carlo ‐calculated dose profiles for different applicators with 10 MeV nominal energy (a) applicator (10 × 10), (b) applicator (15 × 15), (c) applicator (20 × 20) with modification the applicator configuration (d) applicator (20 × 20) without modification the applicator configuration

Table 5 demonstrates the absorbed dose (D_s_) at the surface of the water phantom, the depth of the maximum dose (R_100_), R_90_ (the depth at which the dose reaches 90% of the maximum dose), R_80_ (the depth at which the dose reaches 80% of the maximum dose), and R_50_ (the depth at which the dose reach to 50% of the maximum dose) of the measured and MC calculated PPD. Figure 5 represents the MC calculation and measured dose profiles of 10 MeV nominal‐energy of LINAC at R_100_. In addition, Table 6 presents the R_W50_, F_r_, δ_2_, δ_3_, and δ_4_; where R_W50_ is the width of the profile at 50% central axis value, F_r_ is the difference in beam fringe or penumbra which is defined as the distance between the 90% of maximum and 50% of maximum points on the profile, δ_2_ is the penumbra of primary axis profiles for points in the high dose gradient region – the displacement of isodose curves along the *x* (*y*)‐direction. δ_2_ was measured in regions with dose gradient >2% per mm, where the percentage refers to the percentage of the maximum dose at the depth of the profile. δ_3_ is for the points within the beam but away from the central axis, which has been measured as a percentage difference of the local dose. This includes points just off the central axis to points at 95% of the central‐axis‐dose. In the computation of δ_3_, both primary axis and diagonal profiles were assessed. δ_4_ represents the points on profiles outside the beam geometrical edges, where both dose and dose gradient are low, measured as a percentage difference of central axis dose at the same depth. This quantity is measured at points where the dose is <7% of the maximum value on the profile.^28^

**Table 5:** Measured (Exp), Monte Carlo‐calculated (MC), and the difference (Diff) of the PPD for different applicators with 10 MeV nominal energy.

Applicator	10 × 10 cm^2^			15 × 15 cm^2^			20 × 20 cm^2^		
PPD (%)	Exp	MC	Diff	Exp	MC	Diff	Exp	MC	Diff
D_s_ (%)	85.3	82.8	−2.5	86.10	84.37	−2.73	87.4	85.0	−2.4
R_MAX_ (cm)	2.40	2.30	−0.10	2.40	2.40	0.00	2.40	2.40	0.0
R_90_ (cm)	3.20	3.10	0.10	3.20	3.20	0.00	3.21	3.21	0.0
R_80_ (cm)	3.50	3.50	0.00	3.50	3.50	0.00	3.50	3.50	0.0
R_50_ (cm)	4.05	4.10	0.050	4.05	4.10	0.05	4.10	4.20	0.1

**Table 6:** Comparison of measured and Monte Carlo (MC)‐calculated lateral dose profiles.

Applicator	10 × 10 cm^2^			15 × 15 cm^2^			20 × 20 cm^2^		
	Exp.	MC	Diff	Exp.	MC	Diff	Exp.	MC	Diff
R_W50_ (cm)	5.3	5.4	0.10	8.00	8.00	0.00	10.6	10.5	0.10
F_r_ (cm)	0.90	1.00	0.10	0.90	0.90	0.00	1.00	0.90	0.10
P_90‐10_ (cm)	1.80	1.90	0.10	1.60	1.80	0.20	1.80	2.40	0.60
P_80‐20_ (cm)	1.10	1.10	0.00	1.00	1.00	0.00	1.20	1.10	0.10
δ_2_ (%)	23.7	20.0	3.70	12.0	13.4	1.40	20.9	11.6	9.30
δ_3_ (%)	96.2	95.7	0.50	99.6	100.6	1.00	96.0	98.7	2.70
δ_4_ (%)	5.00	4.60	0.40	1.10	3.00	1.90	5.30	5.90	0.60

## 4 DISCUSSION

From Figures 2 and 3 it was found that the average photon energy of 10 MeV electron beam is about one‐third of the maximum nominal energy. The peak that appears in the energy spectra of incident photons were found at 0.5 MeV, which correspond the electron‐positron annihilation processes, which is similar to previous observations by Mohan et al and Ding et al.^29^, ^30^ Moreover, the peak of electron and positron for all fields is at roughly 10 MeV, which is consistent with the nominal energy. As LINAC utilize the square applicator for electron beam collimation, a greater decrease in the average of beam energy toward the end of the applicator is expected due to the multiple scattering of electrons from the applicator wall.

The differences between lateral field size at the 50% dose level (R_W50_), Penumbra widths, P_90−10_ and P_80−20_ are summarized in Table 6, which obtained using both calculated and measured data. The differences between the measurements and the simulations result in lateral field size at the 50% dose level (R_W50_) were found to be <2 mm.

From Fig. 5, there was a consistency between MC calculation and measurement curves in both dose plateau and the penumbra regions. For all applicators, results of the calculations are under the acceptance tolerance of 3% and 3 mm for both PDD and beam profiles respectively. At the edge of the applicator 20 × 20 cm^2^, the difference between the MC simulation and the measurements data was found to be more than 5% in the dose profile that presented in Fig. 5(d). This variation could be discussed in terms of the thickness and the width of the secondary scattering foil configuration data. However, the MC calculations are too sensitive for the configuration geometry of the scattering foil at the high energy for a large field size such as 20 × 20 cm^2^. Thus, the main reason for this difference between the simulated and measured data for the applicator (20 × 20 cm^2^) is the incorrect scattering foil data supplied by the vendor, Siemens Primus, which ultimately led to the large differences near the field edges in the profile. A similar discrepancy has been observed for a large field size at high energies reported by Bieda et al.^31^ However, the specifications of the treatment head components as supplied by vendors have been found in many cases to be unreliable^32^ and sometimes incomplete.^33^ This has been attributed mainly to the reluctance of vendors to divulge detailed specifications necessary for accurate MC modeling due to the commercial value of the accelerator parts.^34^ Subsequently, in our computation, we endeavor to change the thickness value to reduce the difference and minimize the uncertainty in the dose calculation. These changes were based on trial and error where the secondary scatter foil’s layer 2 thickness has been changed from 0.02 to 0.07 cm and layer 3 thickness has been changed from 0.04 to 0.14 cm. As a result of this change in the secondary scatter foil geometry, a good matching between the simulated and measured data has been obtained.

Table 5 and Fig. 5 are addressed the differences of R_100_, R_90_, R_80_, and R_50_ between the measured and MC calculation for all applicators, which are found <2 mm. The MC calculated depths were mostly equal for all PDDs with measurement depths. As the field size increased, differences between measured and calculated surface doses are increased. However, for all applicators, the differences are <3%, this difference decrease to 0% inside the initial 2.4 mm. Also, the calculated surface doses were lower than measurement for all fields. As preceding, one can observe that the agreement between the MC calculation and measurement of percentage depth dose for all applicator at the nominal energy 10 MeV.

## 5 CONCLUSION

Using MC simulation, characterization of beam spectra and particles statistics were achievable. The PDD and beam profiles were calculated using MC simulation and compared with the corresponding experimental data for different applicator sizes of 10 × 10, 15 × 15, and 20 × 20 cm^2^. Good agreement has been observed between the calculated PDD and the beam profile using MC simulation with the measured data. Better agreement in beam profile for applicator 20 × 20 cm^2^ within 3% and 6 mm was achieved by altering the manufacturer's specifications of the scattering foil. The MC model of the Primus linear accelerator that has been modeled in this study could be utilized as an accurate technique to compute the dose distribution for cancer patients.

## CONFLICT OF INTEREST

The authors declare no conflict of interest.
